# An Overview of Magnesium-Phosphate-Based Cements as Bone Repair Materials

**DOI:** 10.3390/jfb14080424

**Published:** 2023-08-14

**Authors:** Rita Gelli, Francesca Ridi

**Affiliations:** Department of Chemistry “Ugo Schiff” and CSGI Consortium, University of Florence, Via della Lastruccia 3, 50019 Sesto Fiorentino, Italy; rita.gelli@unifi.it

**Keywords:** magnesium phosphates, cements, bone tissue, pastes, setting, porosity, injectability, release, biocompatibility

## Abstract

In the search for effective biomaterials for bone repair, magnesium phosphate cements (MPCs) are nowadays gaining importance as bone void fillers thanks to their many attractive features that overcome some of the limitations of the well-investigated calcium-phosphate-based cements. The goal of this review was to highlight the main properties and applications of MPCs in the orthopedic field, focusing on the different types of formulations that have been described in the literature, their main features, and the in vivo and in vitro response towards them. The presented results will be useful to showcase the potential of MPCs in the orthopedic field and will suggest novel strategies to further boost their clinical application.

## 1. Introduction

The global increase in the population age is nowadays taking place at an extraordinary pace and, particularly in developing countries, it will accelerate in the next few decades: according to estimates from the World Health Organization (WHO), in 2019 the number of people aged 60 years and older was 1 billion; this number will increase to 1.4 billion by 2030 and 2.1 billion by 2050. This large number of elderly people in society, mainly due to an increase in life expectancy, will result in a dramatic increase in a number of pathologies (chronic diseases, disabilities, cognitive decline, hypertension, sleep disorders, etc.) [1]. Osteoporosis is the most common metabolic disease in the elderly, affecting 200 million people worldwide [2] and posing a considerable economic burden to society: the direct annual cost of treating osteoporotic fractures is, on average, USD 5000–6500 billion in Canada, Europe, and the USA alone, not taking into account indirect costs such as disability and the loss of productivity [3].

Besides osteoporosis-related fractures, many other pathologies and damages may affect bone tissue, and new strategies and biomaterials to tackle this major societal challenge are an urgent need. Among the different types of bone biomaterials, bone cements are bone void filling materials that are useful in a variety of orthopedic applications [4] thanks to their moldability and injectability, which allow for minimally invasive surgical procedures [5]. The most widely investigated class of bone cements is undoubtedly calcium phosphate-based cements (CPCs), which have excellent compatibility with the bone matrix given their similarity in terms of their chemical nature: such materials have been extensively studied for several decades [4,6,7,8,9,10], and a number of commercial solutions already exist on the market [11]. CPCs possess many attractive features but, like all biomaterials, they also have some weaknesses, such as a poor degradation rate; unsatisfactory mechanical properties; and, in some cases, acidic setting conditions. To partially overcome these limitations, in the last decade the attention of the scientific community has been directed towards magnesium-phosphate-based cements (MPCs), which have demonstrated a better combination of strength, setting time, and resorption rate than CPCs, while remaining biocompatible [12]. MPCs are already known as rapid repair materials for concrete in the construction field [13], but the interest in them from the biomedical community is rapidly growing.

Recent review articles have already showcased the features and applications of magnesium-phosphate-based biomaterials, highlighting their potential [14,15,16,17,18]. Here, we specifically focus on cement formulations based on magnesium phosphates as bone repair materials, with special attention paid to the various formulations developed and their features, often with a direct comparison to CPCs. The topic of MPCs for biomedical applications was addressed a few years ago by Ostrowski et al. [12] and more recently by Haque and Chen [19], the latter focusing on the biological performance of such materials. The large number of works recently published on the topic and the variety of MPC formulations described testify to the need for an updated work to discuss and compare the recent findings.

In this work, after a brief overview of the main characteristics of bone tissue and its main repair strategies (Section 2), bone cements are discussed in detail in Section 3, focusing on MPCs (Section 4): their main preparation strategies and formulations described in the literature are presented, along with the most interesting properties that make them attractive in the field of bone cements. Section 5 describes the main modification strategies for MPCs, in terms of the different categories of molecules that can be added to the cement matrix to impart functional properties. Section 6 details the biological responses of MPCs, describing the outcomes of both in vitro and in vivo experiments.

The presented results will be useful to showcase the great potential of MPCs in the orthopedic field and suggest novel strategies to further boost their clinical application.

## 2. Bone Tissue and Repair Strategies

**Figure 1:**
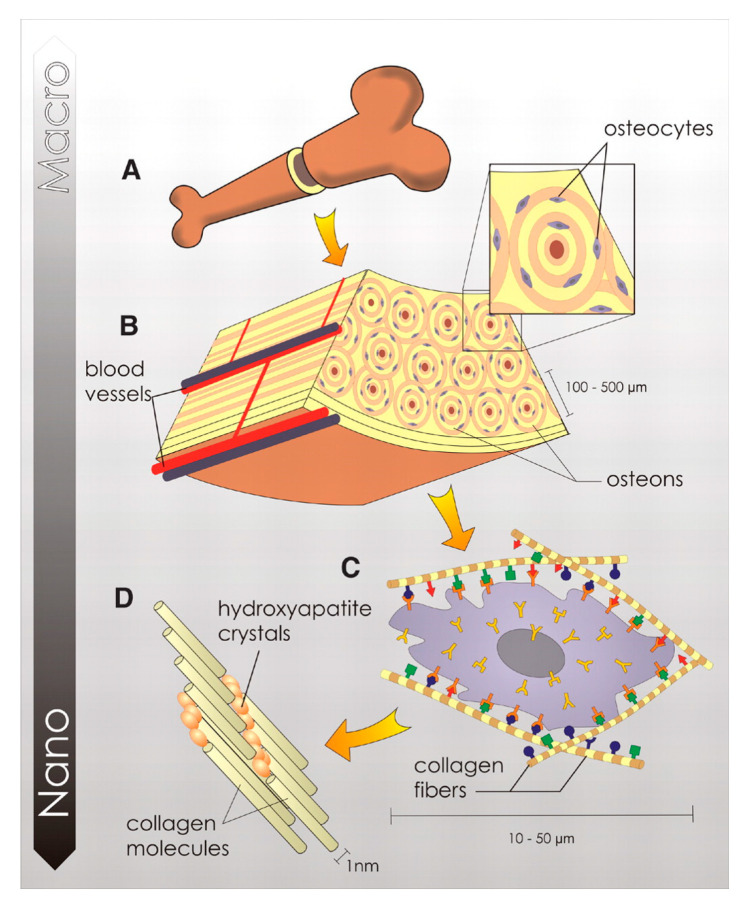
Hierarchical organization of bone tissue at different levels. The macroscale level represents the overall bone shape (A) which, at the microscale, is constituted by osteons (B) and osteocytes coated of cell membrane receptors that respond to specific binding sites (C). At the nanoscale the bone matrix consists of poorly crystalline and Ca-deficient hydroxyapatite platelets and collagen molecules (D). Reprinted with permission from Ref. [22]. Copyright © 2023. American Association for the Advancement of Science.

Bone is a mineralized connective tissue that exerts a number of fundamental functions in our organism, such as locomotion, the support of body structure, the protection of soft tissues, and the provision of a homeostatic calcium and phosphate reservoir. Bone tissue has a hierarchical structure, as schematized in Figure 1, and can be described on different length scales, with each level performing a specific chemical, biological, and mechanical function. The macroscale level represents the overall bone shape. According to the porosity, bone can be distinguished into compact (3–5% porosity) and trabecular (porosity up to 90%) types, with each showing remarkably different mechanical properties [20]. At the microscale, bone structure is described by osteons, which are cylindrical structures about 200 μm in diameter that contain the mineral matrix and living osteocytes, running almost parallel to the long axis of the bone [21]. Each osteon consists of lamellae, which are layers of a compact matrix that surround a central canal called the Haversian canal. This canal contains the bone’s blood vessels and nerve fibers. At the nanoscale, the bone matrix consists of poorly crystalline and Ca-deficient hydroxyapatite platelets (HA, Ca_10_(PO_4_)_6_(OH)_2_) embedded in an organic matrix primarily made of type I collagen.

In spite of its apparently static nature, bone tissue undergoes a constant remodeling process that involves the sequential resorption of bone tissue and deposition of new bone at the same site [23]. In very simple terms, this process can be described in three stages: the initiation of bone resorption by osteoclasts, a transition period from resorption to new bone formation, and new bone formation by osteoblasts [24]. This process occurs thanks to the coordinated actions of the four different types of bone cells: (i) osteoclasts, which dissolve and resorb old bone tissue; (ii) osteoblasts, which form new bone tissue; (iii) osteocytes, which comprise ~90% of bone cells, result from “entrapped” osteoblasts, and maintain bone tissue; (iv) bone lining cells, which are quiescent flat-shaped osteoblasts that cover nonremodeling bone [24,25]. The remodeling process of old or damaged bone is necessary to adjust the architecture and meet the changing needs of the body, as well as to maintain calcium homeostasis and to repair microdamage in the bone matrix [26].

Not all types of bone defect and damage can be fixed by the self-healing capacity of bone tissue: it is estimated that 5–10% of fractures are associated with impaired healing, resulting in delayed union or nonunion [8]. This is a relevant issue for our healthcare system: considering the average number of 8 million bone fractures happening every year in the US, between 400,000 and 800,000 of them will not be able to properly heal [27]. Additionally, in the case of the skeletal reconstruction of large defects due to trauma, infection, tumor resection, or skeletal abnormalities, bone remodeling might not be able to entirely fix the damage. In some cases, bone regeneration is not sufficient because of a compromised regenerative process due to, for example, avascular necrosis or systemic diseases such as osteoporosis and diabetes mellitus. In all these scenarios, natural bone grafts or synthetic biomaterials must be used to bridge the gap before bone regeneration can occur [28,29]. The traditional bone graft strategy is based on autografts (bone from the patient themself), allografts (bone from a human donor), or xenografts (bone from an animal). These approaches are, however, associated with a number of drawbacks, and synthetic materials such as bioceramics, bioactive glasses, metallic materials, polymers, and composites have been developed over the years to improve bone repair processes [28]. In general, bone substitutes should provide both mechanical support and osteo-regeneration, which involves osteoconduction, osteoinduction, and osteointegration [30]. Osteoconduction is the ability to support osteoblasts’ and osteo-progenitor cells’ attachment to the material and allow for their migration and growth within the three-dimensional architecture of the graft. Osteoinduction refers to the graft’s ability to induce the differentiation of cells into bone-forming cells. Osteointegration describes the ability of an implant to anchor to the new bone tissue, with the formation of a bone–implant interface [31]. The formulation of a synthetic bone substitute that meets all these requirements is challenging, and great efforts are being made by the scientific community to develop effective materials.

## 3. Bone Cements

Bone cements are defined as biomaterials obtained from the mixing of a powder phase with a liquid phase, which can be molded and implanted as a paste and can set within the body [32]. They are intended as bone-filling materials rather than bone substitutes [30] and are widely used in different orthopedic procedures, such as vertebroplasty and kyphoplasty [4], and dental implant fixations [33]. Their moldable and injectable nature makes them attractive for minimally invasive surgical procedures, allowing for the filling of irregular bone defects [5]. An ideal bone cement should be endowed with several properties, including biocompatibility, bioactivity, osteoconductivity, injectability, cohesion, the ability to set in physiological conditions, mechanical properties compatible with bone, an appropriate resorption rate, and porosity [6].

Bone cements can be classified according to their chemical composition, namely acrylate-based, calcium-sulfate-based, and calcium-phosphate-based cements. Acrylate formulations mainly consist of polymethylmethacrylate (PMMA). The liquid phase is made of methyl methacrylate, accelerators, and stabilizers, while the powder phase consists of a polymer, a catalyst, and radio-opacifiers. After mixing the two phases, an exothermic polymerization reaction forms PMMA cement [5]. Such materials have been used in clinical settings for over 50 years, after approval was granted by the Food and Drug Administration (FDA) in the 1970s [34]. Nevertheless, the drawbacks associated with their use include the exothermicity of the reaction, which causes tissue necrosis, and the low level of osteointegration due to their nonbioactive and nonbiodegradable nature. Calcium sulfate formulations are based on calcium sulfate hemihydrate (CaSO_4_·0.5H_2_O): when mixed with water, the hemihydrate form is converted into calcium sulfate dihydrate (CaSO_4_·2H_2_O), which is able to set in situ after filling the bone defect [35]. These formulations are osteoconductive, inexpensive, and biodegradable [36]. In addition to PMMA and calcium sulfate cements, the most widely investigated category of bone cements are CPCs, due to their chemical similarity with the inorganic bone matrix. CPCs were introduced by Brown and Chow in the 1980s [37] and approved by the FDA in 1996 [38]. They are typically classified as apatite or brushite (CaHPO_4_·2H_2_O)-based, according to the final phase within the cement matrix [9]. They are obtained by mixing a powder component (one or more calcium orthophosphate salts, such as tri-calcium phosphate) with an aqueous solution: the mixing leads to the dissolution of the initial calcium orthophosphates, followed by the precipitation of either apatite or brushite crystals, according to the reaction pH. Apatite formation is favored at pH > 4.2, whereas brushite forms in acidic conditions [39]. During the precipitation, these new phases grow and entangle, providing mechanical rigidity and resulting in cement hardening. Being made of calcium phosphates, CPCs are characterized by excellent biological properties and offer the possibility of being molded or injected into the bone defect, hardening in physiological conditions, providing support to the bone tissue, and allowing for its regeneration. The detailed description of CPC features was beyond the scope of this work, and interested readers are referred to the many comprehensive reviews that have been published in the last decade on this topic [6,8,9,10,33,40,41,42,43,44]. However, it is important to remark that, notwithstanding the great success of CPCs, which has also led to several commercial products [45], some drawbacks associated with their use have been reported over the years, especially in terms of their poor degradation rate and mechanical properties. In particular, apatite CPCs have often been claimed to have a slow setting time and degradation rate [12]. On the other hand, brushite CPCs are more soluble than apatite CPCs and are hence more quickly resorbed in vivo. However, it has been reported that, upon implantation in animal models, brushite can undergo dissolution–reprecipitation reactions, resulting in phases with lower solubility and thus slowing down degradation and bone regeneration kinetics [46,47]. Moreover, brushite CPCs have very short setting times, and high liquid-to-powder (L/P) ratios must be used to achieve a workable paste, resulting in weak CPCs with limited clinical applications [8].

To overcome these limitations, researchers are currently focusing on alternative materials to CPCs, and a promising category are MPCs.

## 4. Magnesium Phosphate Cements: Preparation and Properties

**Figure 2:**
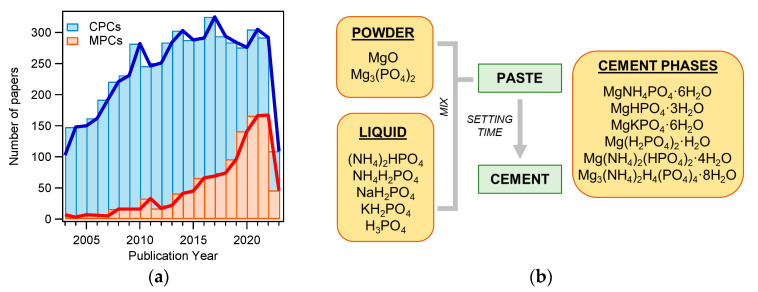
(**a**) Number of publications on the topic “calcium phosphate cement” (CPC) or “magnesium phosphate cement” (MPC) from 2003 to 2023 (Web of Science, updated in May 2023); (**b**) diagram of MPC preparation. The main powder and liquid components are listed, together with the main phases found in the set cements.

MPCs can be defined as cements in which the binding phase is made of magnesium phosphate. The first publication concerning MPCs for applications in the biomedical field dates back to 1995 [48], but such materials were already known as fast-repairing cements for civil engineering applications [49]. The interest in MPCs rapidly grew in the last decade, as can be gathered by the number of publications as a function of the year for MPCs and CPCs: the attention towards the former markedly grew only recently, whereas the number of publications related to CPCs has been relatively constant for the last 10 years (see Figure 2a). From a commercial viewpoint, while dozens of CPC-based products are available, only one company (Bone Solutions, Inc., Colleyville, Texas, USA) is currently commercializing MPCs. Three types of products are available: Mg OsteoCrete, Mg OsteoInject, and Mg OsteoRevive. According to the producers, OsteoCrete is described as a “moldable, injectable magnesium-based bone void filler for trauma/orthopedic applications”, OsteoInject is recommended for sports medicine applications, and OsteoRevive is suitable for posterolateral spine applications. All these formulations are claimed to be moldable; radiopaque; fully synthetic; osteoconductive, with high compressive strength; and endowed with temperature-based setting control, excellent binding characteristics, thixotropic properties, and enhanced bone regeneration.

### 4.1. Preparation of MPCs

MPCs can be prepared using different types of powder and liquid precursors, as schematized in Figure 2b. The powder component often consists of MgO and, less frequently, Mg_3_(PO_4_)_2_ (tri-magnesium phosphate, TMP). The liquid is usually made of an aqueous solution of a phosphate salt (ammonium, sodium, or potassium) or phosphoric acid. Upon mixing, the two components react, forming a viscous and moldable paste. In time, due to dissolution and re-precipitation reactions, the system hardens, forming a compact and solid cement because of the entanglement of the newly formed crystals. The type of phase in the final material changes according to the precursors used and the setting conditions. The different magnesium-phosphate-based phase names, together with the relative solubility and Mg/P ratio, are given in Table 1, while Table 2 shows the precursors and the final phases reported in the literature for MPCs, for applications both as bone cements and in the construction field. Although the latter field was outside the scope of this review, it is interesting to compare these formulations with those used to prepared MPC bone cements and evaluate their differences and similarities.

The data in Table 2 show that the majority of the works carried out so far have used MgO as a precursor powder, which is often reacted with NH_4_H_2_PO_4_ or KH_2_PO_4_ to form STR or KST as binding phases, respectively. TMP has been used to a lesser extent, mostly for reactions with (NH_4_)_2_HPO_4_ resulting in STR. In all cases, a significant amount of reacting powder, either MgO or TMP, was present in the final cement matrix.

Concerning the powder precursors, different factors should be taken into account when using either MgO or TMP. The reactivity of the MgO, for instance, is a crucial parameter, since MPCs can be obtained only using dead burned MgO, i.e., treated at temperatures above 1300 °C. Not only is the calcination temperature important to control the setting process of MgO-based MPCs, but also the deformation ratio of MgO grains, which is the ratio of the experimental specific surface area vs. the theoretical one [56]. This parameter is connected to the disorder of the MgO grains’ surfaces, affecting their reactivity and, as a consequence, the cement setting time. The reactivity of MgO can be easily estimated with the citric acid test, in which a MgO slurry is mixed with a citric acid solution containing phenolphthalein as an indicator [165]. According to the time needed by the formed magnesium hydroxide to neutralize the citric acid solution (indicated by the color change of the solution from white to pink), MgO is classified as having high, medium, or low reactivity. Dead burned MgO is characterized by a neutralization time greater than 900 s. The low reactivity of MgO is necessary to guarantee a homogeneous mix between the powder and the liquid component when preparing a cement; even so, it is often necessary to use a retarder to obtain the desired setting time (see Section 5.1). It should be pointed out that, especially for MPCs applied in the construction field, MgO is typically not pure and can also contain certain amounts of CaO, SiO_2_, Al_2_O_3_, Fe_2_O_3_, K_2_O, Na_2_O, TiO_2_, SO_3_, and P_2_O_5_ [61,62,74,80,95,97,98,131].

TMP, on the other hand, is typically obtained from the calcination of Mg(OH)_2_ and MgHPO_4_·3H_2_O above 1000 °C, followed by grinding and sieving [144,145,148].

The liquid component of an MPC formulation is often an aqueous solution of ammonium or potassium phosphate. When using the former, especially in combination with MgO, the release of ammonia and the highly exothermic reaction that occurs during the hydration process can be drawbacks. In this case, replacing the ammonium with potassium salts, which do not result in the above problems, is a viable option [95,166].

The preparation procedure is similar for all MPCs, though a difference according to the type of precursor can be found: MgO is often mixed with the phosphate salt as a solid, and water is then added to the formulation; in this case, the w/c (water/cement), w/b (water/binder), or w/s (water/solid) ratio is specified. When TMP is used, the phosphate salt is first dissolved in water, and this solution is then mixed with the TMP powder: the proportions between the two components are given as the P/L (powder/liquid) ratio. Regardless of the precursor and the additional materials included in the formulation, the cements are prepared by mixing, molding, and hardening, often in controlled conditions of relative humidity.

**Figure 3:**
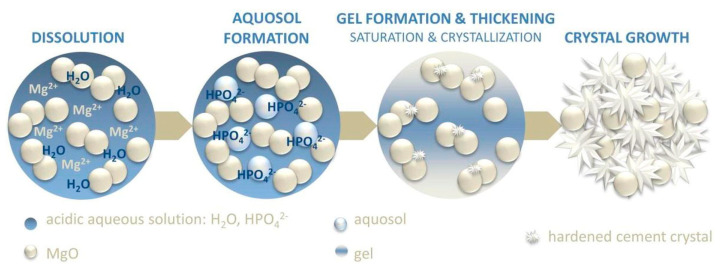
Schematic of the setting mechanism of MgO−based MPCs. Reprinted with permission from Ref [14]. Copyright © 2023. Elsevier.

As we previously mentioned, in MgO-based formulations, the phosphate salt is often included as a solid within the powder component. When the mixture comes into contact with water, the phosphate salt immediately dissolves, leading to the release of protons and decreasing the pH of the solution [19]. According to the mechanism proposed by Wagh and Jeong [167], MgO slowly dissolves from the edges of the grains, leading to the formation of an intermediate aquosol. Afterwards, the acid–base reaction with the phosphate groups in the solution results in a gel that forms a layer on the surface of the undissolved MgO grains. In time, this gel thickens and hardens due to the growth of the crystalline binding phases, leading to the final hydration product (see Figure 3). It is worth noting that some transient phases among those reported in Table 1 might form during this process [168]. The reaction mechanism occurring when using TMP as a precursor powder has been investigated to a lesser extent. In a similar way, it is believed that the reaction occurs on the surface of the TMP grains, where the Mg^2+^ ions react with phosphate and ammonium ions from the aqueous component, resulting in the precipitation and entanglement of the binding phases [159].

Concerning the application areas, the data in Table 2 demonstrate that MgO is mainly used for cements applied in the construction field, whereas TMP is favored for preparing bone cements, sometimes taking advantage of 3D printing to assemble them as porous scaffolds [140,145,146,150,152,155]. Despite the use of MPCs in the construction field being outside the scope of this review, we mention here that such materials are typically exploited for the rapid repair of concrete—not as substitutes for Portland cement—due to their rapid setting, high early strength, corrosion resistance, low shrinkage, good adhesion with concrete substrates, and abrasion resistance. Applications for radioactive waste stabilization and solidification have also been reported [95].

### 4.2. Properties of MPCs

**Figure 4:**
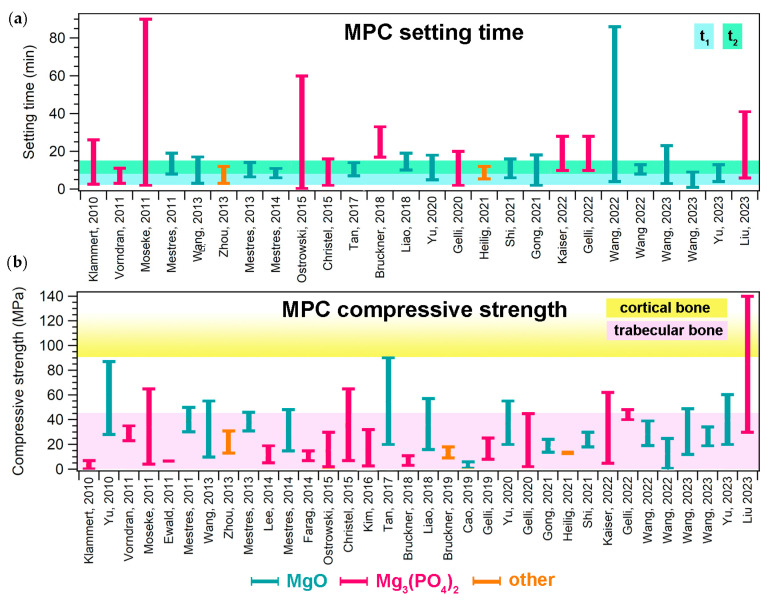
(**a**) Setting time ranges given in the literature for MPCs designed for biomedical applications; for each paper, the bar ranges between the minimum and maximum values reported for a specific formulation. The colored area indicates the ideal t_1_ and t_2_ ranges, according to [7]. (**b**) Compressive strength ranges reported in the literature for MPCs designed for biomedical applications; for each paper, the bar ranges between the minimum and maximum values reported for a specific formulation. The colored areas indicate the compressive strength ranges of trabecular (2–45 MPa) and compact (90–230 MPa) bone [8]. In both graphs, the different bar colors indicate the precursor powder: light blue (MgO), pink (Mg_3_(PO_4_)_2_, and orange (others). The references mentioned in the figure are the following: [33,57,58,60,63,79,81,97,108,109,117,118,132,133,134,137,138,139,140,141,144,145,146,147,148,149,150,151,152,156,157,158,161,162].

Many properties make MPCs attractive in the field of bone cements. One of these is their setting time, which is typically in line with clinical needs. The setting time of a bone cement can be roughly defined as the time needed for the cement paste to become strong enough to resist an applied force [6]. This parameter can be measured with different tests, such as the Gillmore or Vicat tests, the former being the most commonly used for MPCs designed as bone cements. During the Gillmore test, the initial (t_1_) and final (t_2_) setting times of the paste are determined: t_1_ is the time needed for the cement to bear a thin and light (113 g) needle without appreciable indentation, while t_2_ is the time needed for the cement to bear a thick (454 g) and heavy needle without appreciable indentation (ASTM C266-21). There is clinical significance behind these values, as the cement paste should be implanted before t_1_ and the wound should be closed after t_2_ [7]. It is therefore important that the paste sets slowly enough to allow for mixing and molding/injecting in the bone defect. At the same time, it should not set too slowly, so that the wound can be quickly closed and the set cement provide the needed mechanical support. In the literature, it has been suggested that the ideal ranges for t_1_ and t_2_ are 3 ≤ t_1_ < 8 min and t_2_ ≤ 15 min, respectively [7]. As can be observed from the data reported in Figure 4a, most MPC formulations are able to set within the desired time frame, irrespective of the precursor used. The setting time depends on several parameters, namely the powder’s characteristics (size, reactivity, and specific surface area); the aqueous solution concentration; the P/L ratio; the temperature; and the humidity [12]. In some cases, if the modulation of these parameters is not sufficient or possible, the setting time of the paste can be extended through the inclusion of retarders (see Section 5.1).

Another important feature of bone cement pastes is their cohesion, which is defined as their ability to harden in a fluid without disintegration [169]. The cohesion is connected to the “anti-washout ability” of the cement, which is typically determined in a dynamic environment, while “cohesion tests” are carried out in static conditions [6]. Cohesion is an important feature, since MPCs, when used to fill a bone defect, will come into contact with biological fluids before they are completely hardened: an ideal cement should then be able to retain its shape and harden even when in contact with aqueous media. This property has been well addressed in the CPC literature [170,171,172,173,174], whereas for MPCs it is often neglected, and very few publications have discussed this aspect. While developing antimicrobial MPCs for dental applications, Mestres et al. determined their cohesion time as the time needed by the cement to no longer disintegrate when immersed in an aqueous solution by visually inspecting cement disks soaked in distilled water [63]. They found that less than 7 min was required by all the tested formulations to attain the cohesion time. Polymeric additives such as chitosan have been reported to improve the cohesion of pastes when in contact with water [60]. Yu et al. found that an intermediate concentration of carboxymethyl chitosan prevented paste disintegration upon injection in Ringer’s solution [109], as well as when used in combination with alginate [138]. Foaming agents can also help in improving anti-washout resistance [117]. Recently, Liu and colleagues reported remarkable results in the direct extrusion of a TMP-K_2_HPO_4_ cement in simulated body fluid without the use of additives: the cement, after shaking for 15 min at 37 °C, did not show any significant powdering or dissolution, proving its excellent anti-washout resistance [160]. Heilig et al. recently showed that MPCs based on a combination of TMP and MgO reacted with phytic acid also displayed notable cohesion properties, as they could be manually injected into water without the appearance of any streaks on the water surface [162].

**Figure 5:**
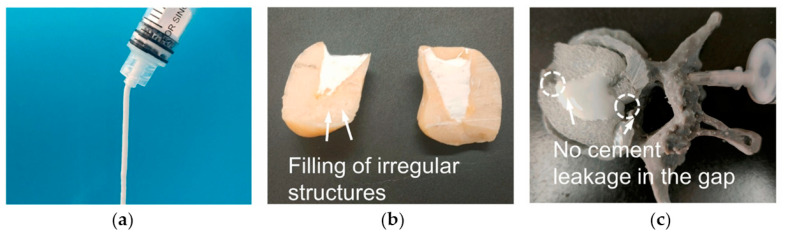
(**a**) Injectability of an MPC paste. (**b**) Filling performance of the MPC paste in irregular models. (**c**) Filling and leakage performance of the MPC on a 3D-printed human vertebral body model. Adapted with permission from Ref. [160]. Copyright © 2023. Elsevier.

The cohesion is strictly connected to the injectability of a cement paste. The injectability of MPCs is one of their most appealing features from a clinical perspective, as it allows one to perform minimally invasive surgeries, which are in general associated with reduced pain, shorter hospital stays, and fewer complications compared to traditional surgeries. In addition, the possibility of injecting a paste rather than implanting a block or solid materials allows in principle for improved bone-to-implant contact [8]. In Figure 5, an example of an MPC application in a bone defect, highlighting the importance of the injectability, is provided.

Injectability depends on both the paste properties and the injection parameters. When assessing this parameter, the clinically relevant conditions in which bone cements are applied should be kept in mind in the design of the experiment: the needles used in percutaneous surgeries such as vertebroplasty or kyphoplasty usually range from 13- to 8-gauge (internal diameters of 1.80–3.43 mm) and from 10 to 15 cm in length [175]. The injectability of MPC pastes is a property frequently evaluated in the literature. Typically, the % of extruded paste with respect to the amount initially loaded in the syringe is calculated, and the injectability % is obtained. The injection can be performed manually [109,158] or in controlled conditions, using specific loads and testing machines [58,160,162]. Mestres et al. compared Na-MPCs, NH_4_-MPCs, and a mixed system, observing that Na-based MPCs had a higher injectability % [58]. The inclusion of carboxymethyl chitosan in the MPC has been reported to extend the injectability time, in a concentration-dependent fashion, as well as the inclusion of alginate [138]. The injectability % and the injection time both increased when including a foaming agent based on calcium carbonate and citric acid [117]. In TMP-based formulations, the work of Moseke et al. showed that the partial replacement of (NH_4_)_2_HPO_4_ with (NH_4_)_2_citrate led to an improvement in the injectability % [144]. MPCs obtained from the combination of both TMP and MgO also displayed excellent performances in terms of injectability %, reaching 100% at forces below 300 N [162]. In MPCs, a low injectability % can be due to several reasons, namely a short setting time (i.e., the paste hardens too fast during the extrusion process); a high viscosity; and phase separation (or filter pressing) phenomena. In the early stages of cement formation, the unset cement consists of a powder component dispersed in a liquid. If, upon injection, the liquid phase travels at a faster rate than the powder particles, the extrudate will contain a much higher liquid content than the paste initially loaded into the syringe, eventually leading to a cement with markedly different properties. Phase separation phenomena are well-established for CPCs [9], whereas in the MPC literature, the characteristics of the extruded pastes are often neglected.

While the properties of MPC pastes are fundamental for the administration of the material in the body, the characteristics of the set cements are of course crucial to determine their performance when implanted in bone in terms of biocompatibility, mechanical integrity, dissolution behavior, and the interplay with bone cells.

Concerning the mechanical properties of MPCs, it is important to remark that such materials are classified as unsuitable for load-bearing applications [12,14]. They are in fact intended not to replace large regions of bone to create grafts, but rather to provide a support to repair small bone defects and damage. Nonetheless, it is important that in this process they are well integrated in the bone, displaying suitable mechanical properties to provide support. The mechanical property that is most frequently evaluated in the MPC literature is compressive strength. Figure 4b shows the ranges of compressive strength that have been reported for MPCs in the biomedical field, compared to those associated with cortical and trabecular bone: the majority of the MPCs reported in the literature could match the compressive strength of trabecular bone (2–45 MPa) but did not reach 90 MPa, which is the minimum value for cortical bone. [8]. We should remark that the values reported for MPCs in Figure 4b are not easily comparable, as the different values might have been due to not only the different formulations, but also the variations in the setting environment of the samples and the testing conditions, which drastically affect the strength of cements. One of the most attractive features of MPCs, compared to CPCs, it their ability to reach these strength values in relatively short times (i.e., high early strength) [14,57]. Strategies to improve the mechanical properties of MPCs could take inspiration from the inclusion of additives used for this purpose in CPCs or PMMA-based cements, including glass, Ti and C fibers, and graphene oxide [34,176,177].

**Figure 6:**
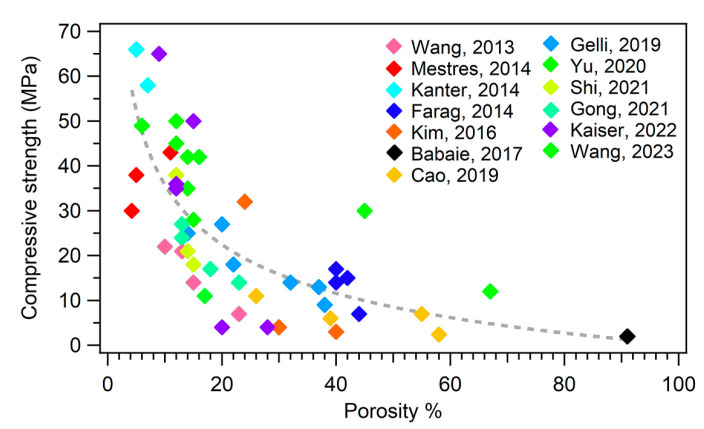
Plot of the compressive strength as a function of porosity %. The different colors of the markers refer to different publications, as reported in the legend. The dashed line is a guide for the eye. The references mentioned in the figure are the following: [46,58,69,81,108,109,118,132,137,148,150,152,157].

The mechanical properties are intimately connected to the microstructure and porosity of an MPC. It has been well established that a strategy to improve the mechanical properties of MPCs, and cements in general, is to reduce their porosity. Figure 6 shows a plot of the compressive strength as a function of the porosity % of different MPC formulations extracted from the literature.

One might therefore think that minimizing the porosity of the material could be an effective strategy to boost the performance of an MPC. However, a decrease in the porosity of a cement to enhance the mechanical properties might be detrimental for the biological response of the material: a porous cement is in fact fundamental to favor bone ingrowth and cement degradation, which is connected to in vivo resorbability.

In principle, porosities on different length scales should be present in cements to achieve favorable bone integration. This aspect was recently reviewed by Lodoso-Torrecilla et al. for CPCs [8], highlighting that these materials possess an intrinsic microporosity resulting from the entanglement of the crystals formed during the setting process. This microporous structure enables the flow of biological fluids and protein adsorption but does not allow for bone cell permeation and ingrowth. The pore size generally considered adequate for bone regeneration is at least 100 μm, as smaller pores may result in the formation of unmineralized or fibrous tissue and may not allow for the growth of blood vessels [178,179]. To achieve this, macropores must be induced on purpose in the MPC matrix. In the literature, few attempts have been described for the preparation of macroporous MPCs: zinc powder [59], also in combination with a chemical foaming agent [73]; sodium bicarbonate [104]; and protein-based foaming agents [103] have been used, even though none of these materials were designed for application in the biomedical field. Macroporous MPCs for orthopedic applications have been obtained using biodegradable Mg particles as porogens during cement setting [69], polyurethane foams [143], and gelatin microparticles [158].

**Figure 7:**
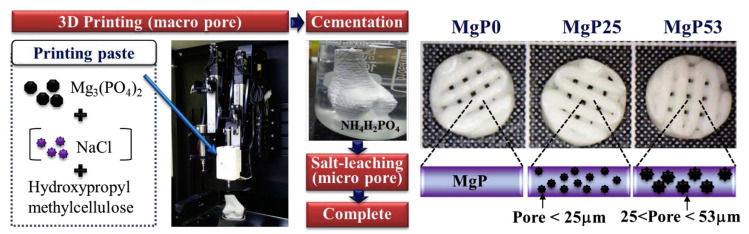
Schematic diagrams showing the fabrication of MPC scaffolds with different pore structures using 3D printing, NaCl leaching, and cement reaction. Reproduced with permission from Ref. [150]. Copyright © 2023. Elsevier.

An effective solution to obtain pores of different sizes in MPCs consists in the inclusion of NaCl granules of various granulometries in 3D printed scaffolds: additive manufacturing allows for the creation of scaffolds with a tailored size and the building of macro-sized pores > 300 µm. Salt leaching leads to the dissolution of NaCl grains, leaving behind a porous structure with pores below 25 µm and between 25 and 53 µm produced using different sizes of NaCl templates (see Figure 7) [150].

An increase in porosity is inevitably connected to a decrease in the mechanical properties (see Figure 6), so one should carefully design the formulation parameters of MPCs according to one’s specific needs.

The enhancement of cement porosity is, for instance, important when the material needs to be quickly resorbed in vivo. The fast degradation rate of MPCs is one of their main advantages when compared to CPCs. Magnesium phosphates are typically more soluble than their calcium counterparts, and this is often described as one of their most appealing features. In fact, MPCs are not expected to endure when clinically injected: on the contrary, they should be resorbed and replaced over time by the body’s own regenerated tissues, providing temporary support. The dissolution timeframe should ideally match the body’s bone reforming ability. In fact, the overly rapid degradation of the MPC might induce some side effects, as in the initial stages the cement should provide structural integrity to the bone defect, allowing for the healing mechanisms to take place. In vivo degradation can take place through two pathways: (i) passive degradation by the dissolution of the cement matrix into the biological fluids, and (ii) active degradation due to cellular activity. The first pathway is often assessed by incubating cements in aqueous fluids, such as PBS, SBF, or Tris-HCl buffer at pH 7.4, and evaluating their weight loss. Although such experiments do not directly translate to in vivo degradation rates, they are a valuable indication of the comparative rate of dissolution between different material compositions [12]. Even though mixed CPC–MPC cements were outside the scope of this review, it is useful to provide a few examples to demonstrate the different dissolution behaviors of the two materials. For instance, Wu et al. developed mixed CPC–MPC formulations and studied their degradation in SBF over 90 days [180]. They found that the CPC lost less than 5% of its initial weight, while the MPC reached a weight loss of about 60%. Interestingly, the intermediate formulations showed intermediate dissolution rates, and the mixed cement provided faster and more effective osteogenesis in the defect area compared to the pure CPC. Another work from Wu et al. provided similar results [181]. Concerning pure MPCs, Mestres et al., comparing cements prepared using either sodium or ammonium phosphate as the aqueous phase, showed that upon immersion in PBS for 60 days, the weight loss at the end of the experiment was 4.3%, 5.2%, and 2.4%wt for NH_4_-MPC, Na-MPC, and NH_4_ + Na-MPC, respectively [58]. Higher dissolution values were recently reported by Yu et al. [138], who incubated MPCs modified with carboxymethyl chitosan and alginate in saline solution for 28 days and observed a weight loss % between ~15% and 18%, depending on the polymer concentrations in the cements. MPCs recently obtained by mixing TMP with potassium phosphate and incubated in Tris-HCl buffer (pH = 7.4) showed a weight loss % of about 12 % after 28 days of the experiment [160]. An interesting work from Kaiser et al. [157] compared the dissolution behavior of MPCs based on STR and KST with a reference of calcium-deficient hydroxyapatite: after incubation for 18 days in PBS, the STR and KST cements lost 1% and 8% of their initial weight, respectively, while the hydroxyapatite reference did not show any sign of degradation. Similar results were obtained by Ewald et al., who compared the weight loss of cements made of STR with two calcium phosphate references, brushite and calcium-deficient hydroxyapatite, upon incubation in PBS for 20 h: the hydroxyapatite-based material did not dissolve, while both the brushite and STR showed a cumulative dissolution normalized to the cement surface of approximately 2 mg/cm^2^ and 8 mg/cm^2^, respectively [141]. On the other hand, Cao et al. compared the degradation % of STR, NEW, and brushite-based scaffolds in PBS, showing that NEW and brushite slowly degraded over time, reaching ~22% and ~10% weight loss after 4 weeks, respectively [81]; STR was unexpectedly found to gain about 22% of its initial weight, and the authors attributed this result to the formation of Mg(OH)_2_ due to the reaction between the MgO precursor present in the cement and the water in the PBS.

Lee et al. studied the degradation of MgP scaffolds prepared using 3D printing [146]: PBS was used as the incubation medium, and after 20 days the materials retained 92% of their initial weight. Recently, Wang et al. observed that the presence of different clay minerals in MPC formulations modified their dissolution behavior in SBF and PBS at 28 days [137]. When amorphous magnesium phosphate was used as a powder precursor, the cements soaked in SBF for 8 days lost a considerable amount of weight, ranging from about 24% to 50%, depending on the quantity of PVA included in the formulation [164]. High dissolution values have also been reported in MPC–ZIF8 composites, which showed a weight loss between 39 and 46% upon incubation for 28 days in Tris-HCl buffer [133]. Similar experimental conditions (Tris-HCl buffer for 28 days) led to different results in studies by Gong et al., who observed a weight loss between 12 and 15 % in MPCs modified with oxygen-carboxymethyl chitosan [132], and Yu et al., showing a similar level of degradation for MPCs with carboxymethyl chitosan [109]. Wang et al. implemented the same experimental conditions and recently reported a weight loss between 15 and 22 % in MPCs prepared with a foaming agent [117], similar to the work of Shi et al. [108].

Such variations in the observed dissolution/degradation values can be attributed to not only the different formulations, but also the variety of experimental conditions used (the incubation medium, duration of the experiment, refreshing of the incubating solutions, etc.).

The study of the dissolution/degradation of cements in water is often accompanied by a study of the release kinetics of the constituent ions [58,109,138,146,157].

Finally, it is worth recalling that the slow degradation rate attributed to CPCs is mainly restricted to the apatitic ones, whereas brushite-based CPCs can dissolve more easily [182]. However, upon the dissolution of brushite, low-solubility phases might re-precipitate in vivo [46,47]. The presence of Mg^2+^ in cements prevents this issue, as Mg^2+^ ions hamper the crystallization of apatitic phases [183].

Last but not least, the biocompatibility of MPCs is indeed a fundamental parameter. This aspect has been well documented in the literature, on the premise that magnesium phosphates are made of biocompatible components and are naturally present in our organism as components of calcification. This factor will be explored in detail in Section 5 of this review.

In summary, MPCs possesses many attractive features for the development of bone cement and are endowed with a higher strength, porosity, and resorption rate than CPC implants, while preserving good biocompatibility [19]. It is also evident that the enhancement of a specific property might often be detrimental to others, and researchers should carefully design materials according to their specific needs.

## 5. Modifications of MPCs

The data reported in Table 2 show that in the literature, MPCs have been prepared with a great variety of formulations in order to improve specific features (injectability, porosity, compressive strength, and so on). Besides the use of different powder precursors (MgO or TMP) and liquid precursors (aqueous solutions of mainly ammonium, sodium, or potassium phosphates), a variety of ingredients have been used as additives to impart specific functional properties to the cements. In the following paragraphs, the most important categories will be summarized.

### 5.1. Retarders

Retarders are used to increase the setting time and reduce the intensity of the exothermic reactions during the initial setting and hardening stages of cement pastes. The short setting time is in fact one of the most characteristic and appealing features of MPCs as bone cements, as we outlined in the previous section. It is important to keep in mind that, although MPCs display an intrinsically short setting time, the possibility of modulating this parameter would be fundamental to open up new possibilities in the use of such materials. Moreover, a rapid setting time is often connected (especially in MgO-based formulations) with an abrupt temperature increase due to the heat generated from the setting reaction. The possibility of slowing down the temperature rise during setting is thus fundamental for the application of MPCs in the orthopedic field, as excessive heat release might cause tissue necrosis and limit their use [184].

For this purpose, the role of additives that could prolong the setting time and in turn increase the workability and moldability of cements has been investigated in the literature. The data summarized in Table 2 show that by far the most widely investigated retarder for MPCs is borax (Na_2_B_4_O_7_·10H_2_O)/boric acid (H_3_BO_3_), in both the biomedical [57,58,63,77,126,135] and (particularly) construction fields [59,62,64,65,66,67,68,70,71,72,73,74,75,76,77,80,82,83,84,85,86,87,89,90,91,92,101,102,104,107,112,113,116,119,120,124,128,129,130,131,136]. Borax was in fact traditionally used to delay the setting time of MgO-based MPCs, and its setting mechanism has been well investigated in the literature, from both an experimental and a theoretical perspective [56,96,100,167,168,185]. Its efficacy in retarding the setting of TMP-based MPCs was reported only recently [159]. Irrespective of the precursor powder used, borax can delay the setting time of MPCs from several minutes to a few hours, depending on the concentration and other formulation parameters. It is important to remark that some concerns about the toxicity of borax for reproduction have recently been raised [186], and the optimization of different retarders has been sought.

Another retarder traditionally used for MgO-based MPCs is sodium triphosphate (or tripolyphosphate), Na_5_P_3_O_10_ [65,67,70,71,75,78,83,107,125]. This salt has been used both alone and in combination with borax (see Table 2) and is able to delay the setting of MPCs. Even though its mechanism of action has been poorly investigated in the literature, it is known that Na_5_P_3_O_10_ is very effective in chelating Mg^2+^ ions, being in fact commercially used as a detergent builder and, in particular, as a water softener. This property causes an interaction with Mg^2+^ during the cement setting, delaying the formation of the binding phases.

Other salts are reportedly able to slow down the setting process of MPCs: the inclusion of Zn(NO_3_)_2_ in proportions ranging from 1 to 4% by weight with respect to the solid was observed to delay the setting of the MPC, slow down the increase in the pH, and delay the hydration exothermic peak of the MPC, while decreasing its compressive strength [116]. Also, CaCl_2_·6H_2_O can increase the setting time from about 5 min to 30 min when present in proportions ranging from 0 to 2.5% [187]. This additive was determined to be not very effective in reducing the temperature increase during setting, since 55 °C was reached even at the highest CaCl_2_ concentration tested; however, it did not hamper the compressive strength of the material. Recently, the retarding effect of Al(NO_3_)_3_ was thoroughly investigated in a theoretical and experimental study dealing with MPCs based on KST [136]. Its effect on the hydration of the cement paste was examined, and it was found that three processes contributed to the retarding effect, i.e., the acidic nature of aluminum nitrate, which decreased the initial pH; the early precipitation of an amorphous K-aluminophosphate; and a salt effect due to the presence of soluble nitrate.

Some organic acids have also been reported as retarders for MPCs, in particular, acetic [62,64,67] and citric acid [73,111,117,188]. Besides the increment in the setting time, the inclusion of citric acid has also been reported to positively impact the compressive strength of cements [188]. Ammonium citrate has also been included in some MPC formulations [144,156], partially replacing ammonium phosphate as a binding liquid. Citrate was also able to increase the injectability of cements [144], and its inclusion in MPCs might be beneficial in terms of bone regeneration processes. Citrate inclusion in bone biomaterials has in fact demonstrated many interesting outcomes, affecting gene expression [189] and participating in the treatment of osteopenia and osteoporosis [190,191].

Glucose is another molecule that has shown retarding effects when included in KST-based MPCs. When included in these formulations, glucose was able to extend the setting time from 7 to 14 min, depending on the concentration, and markedly decrease the temperature of hydration: in particular, in the absence of glucose, the maximum temperature reached during setting was about 60 °C, while the addition of 12 %wt glucose prevented the temperature rising above room temperature [97].

Phytic acid (inositol polyphosphate) was used as an innovative binding phase to prepare MPCs [147,161,162,163]: this molecule contains six phosphate groups, which could react with MgO-TMP, forming NEW as a cement binding phase. It was observed that the increase in phytic acid concentration resulted in an enhancement in the setting time of the cement from 11 to 16 min, representing a timeframe of interest in the bone cement field [147].

Polymers can also modulate the setting time of cements: Zárybnická et al. recently reported that polyvinyl alcohol cross-linked with glutaraldehyde could extend the working time of cement with a reduction in the overall reaction rates and the rate of heat evolution [192].

An alternative approach to controlling the setting process of MPCs is to use pre-reacted MgO as a starting powder [184].

In summary, a variety of retarders can be used to modulate the setting process of MPCs, and when a safer alternative to borax should be used, a number of substitutes can be explored. It is worth noting that most studies have explored the effect of retarders on MgO-based MPCs, and there is great room for improvement in the field of retarders for TMP-based formulations.

### 5.2. Biorelevant Molecules

Besides retarders, in the MPC literature, some reports have described the inclusion of different types of molecules to impart functional properties to cement. This technique has been well developed in CPCs, in particular, in the field of drug release [10,166]. Bone cements can in fact be formulated to include within the matrix an active molecule to be released upon contact with biological fluids or in vivo implantation. The potential of this method has been far less extensively explored in MPCs, given that few reports have described the loading and release of drugs in such materials. It was recently pointed out that, so far, no data on the use of MPCs as drug carriers have been reported in the literature [166]. A closer look at the topic shows that few studies concerning the loading of biorelevant molecules in MPCs can be found in the literature. In this context, Lee et al. studied the release of lysozyme from a 3D-printed MPC scaffold [146]. Lysozyme was chosen as a model protein due to its isoelectric point and molecular weight, which are similar to those of the typical growth factors used in tissue engineering. This protein was included in MPC scaffolds using two strategies, either directly blending the protein with MgP powder before cement formation or incubating the prepared scaffold in a protein-rich solution. The differences in loading efficiency and release between the two preparation techniques were assessed, demonstrating the possibility of obtaining MgP scaffolds as effective drug delivery carriers that can maintain a high loading efficiency and bioactivity. A study from the same group showed that the release of lysozyme from a 3D-printed MPC scaffold was dependent on the amount of gelatin included in the formulation [152].

In a recent study, an anti-osteoporotic drug, alendronate, was included in a TMP-based MPC [158]. The drug was loaded into gelatin microparticles of different sizes, which were used as templating agents for the creation of macroporous cements, and its release kinetics were investigated. Kim et al. incorporated as a bioactive molecule a novel indene compound that showed osteogenic properties in a 3D-printed MPC scaffold [155]. Citrate itself, as we pointed out in Section 5.2, can be considered as a biorelevant molecule, given its role in bone metabolism [193]. Despite being often used as a retarder, its release from MPCs when incubated in water has rarely been reported [156].

The above-described examples clearly demonstrate that the loading of bioactive molecules in MPCs is a relatively unexplored field that is definitely worthy of investigation. The inclusion of active molecules in these materials, for instance, antibacterials, could further boost MPC application in the orthopedic field and open up new possibilities and application fields.

### 5.3. Polymers

Polymeric additives have often been included in MPCs for biomedical applications to achieve specific properties. One of the most commonly exploited polymers (especially in TMP-based MPCs) is HPMC (hydroxypropylmethylcellulose), due to its rheology-modifier properties. This modified cellulose is widely used in the food, cosmetic, and pharmaceutical industries, as well as in detergents and paints. In MPC formulations, it has often been used when the preparation procedure involves 3D printing [145,146,150,152,155]. Vorndran et al. added 1% HPMC to the powder mixing before printing in order to prevent the spreading of the liquid, thus allowing for the printing of dimensionally consistent samples [145]. Farag et al. combined HPMC with various amounts of gelatin in the preparation of their 3D-printed MPC and demonstrated that gelatin could improve the compressive strength of the obtained scaffold [152].

An alternative polymer used for the 3D printing of MPCs is Pluronic F127, a block copolymer of poly(ethylene oxide) and poly(propylene oxide) [81]. An aqueous solution of this polymer was used as a binder and mixed with light MgO to create an injectable paste ink that could be used to create a scaffold for immersion in an ammonium phosphate solution in order to harden and create an MPC-based material.

Besides the characteristics of the material during 3D printing, the purpose of polymer inclusion in MPCs is often motivated by a need to improve the water resistance and injectability of MPC pastes. Chitosan, a biopolymer derived from chitin deacetylation, has been used for this purpose [60]. It was hypothesized that the polymer could interact with the surface of the MPC hydration product, inhibiting the contact between H_2_O and the hydrates, thus reducing the precipitation of phosphates and improving the water resistance. Modified chitosan, namely carboxymethyl chitosan (CMC, pure [109] or in combination with alginate [138]) and oxygen-CMC [132], have also been used. CMC markedly improved the characteristics of the paste, extending the injectability timeframe for the formulations and endowing the material with superior anti-washout resistance [109]. Increasing the amount of CMC also increased the setting time and decreased the temperature reached during the setting process.

Recently, poly(N-isopropylacrylamide) and mucin were tested as additives for MPC formulations to improve injectability and anti-washout resistance [194]. It was demonstrated that mucin, a biopolymer derived from the mucus layer of the intestine that is known for its lubricant properties, could improve the injectability of MPC pastes and decrease the dissolution of the material when extruded in Ringer’s solution.

Polyvinyl alcohol (PVA) has been used in amorphous magnesium-phosphate-based cements to tune many of the properties of the material: it was found that formulations with a 15% PVA content and a P:L ratio of 0.5 showed the best combination of setting time, no exothermicity, and acceptable biodegradation and compressive strength values [164].

The inclusion of polymers in MPCs is not restricted to the improvement of the material properties, as it can also influence the response of the cement toward cells. In this regard, MPCs have been prepared with chondroitin sulfate, a glycosaminoglycan present in the extracellular matrix and at the cell surface that is known for its osteogenic potential and high biocompatibility [108]. Shi et al. showed that chondroitin sulfate prolonged the setting time of the cements; increased their compressive strength; and, most importantly, promoted in vitro pre-osteoblast cell proliferation, attachment, and differentiation, enhancing bone formation surrounding the implants in vivo.

Overall, a small number of polymers has been tested as additives for MPCs in orthopedic applications, and many more could be explored to improve their injectability, rheological properties, and response to cells.

## 6. Biological Performance

Mg is one of the most important elements present in our body: it is the fourth most abundant ion in mammalians behind Na^+^, K^+^, and Ca^2+^, and it is the second most prevalent intracellular cation after K^+^ [14]. The estimated total amount of Mg in the human body is 20–28 g, of which ~65% is located in bone tissue and about 32% is complexed with nucleic acids and proteins [15]. Table 3 shows the average Mg content in the different tissues of an adult human [16].

Magnesium plays a variety of important roles in cells, such as the regulation of the calcium and sodium ion channels, the stabilization of DNA, and the stimulation of cell growth and proliferation; moreover, it also acts as a cofactor and catalyzer for many enzymes. Magnesium has a multifunctional role in bone growth and regeneration. It exerts both direct and indirect effects between connecting bone, vessel, nerve, and immune systems, generating potential for functional bone regeneration. These cellular and molecular mechanisms have contributed to the enhanced healing of long bone fractures in animal models, including osteoporotic animal models [195]. Interestingly, it has been shown that a Mg deficiency leads to a number of health problems, including low bone mass, impaired bone growth, osteoporosis, and increased skeletal fragility [196]. In vitro studies showed that Mg^2+^ release stimulated osteoblast differentiation and inhibited osteoclast formation in a dose-dependent manner [14]. It is, however, important to always keep in mind that excessive concentrations of any ion can be cytotoxic to cells (for Mg^2+^, 20 mM has been reported as a cytotoxic dose [197]), and the fine-tuning of Mg^2+^ release from a biomaterial is important. For instance, Wu et al. observed in human osteoclasts that cell proliferation and differentiation increased or decreased depending on the Mg^2+^ concentration [198].

In the field of MPCs, their biocompatibility and interaction with cells is of critical importance for the clinical application of these materials. Their dissolution and subsequent Mg^2+^ release might in fact affect cell behavior and the ultimate success of the clinical application. Although the field of MPCs as bone cements is relatively new, several works have already demonstrated their excellent response toward both cells and in vivo implantation. These two aspects are explored in the following paragraphs.

### 6.1. In Vitro Experiments

The cytocompatibility of MPCs has often been evaluated in the literature along with the material properties. Concerning the precursor powders that can be used, Ostrowski et al. assessed the osteoblast and osteoclast viability of amorphous and crystalline TMP [199]. The more soluble amorphous TMP showed higher levels of osteoblast viability and differentiation with respect to crystalline TMP: such increased osteoblast viability was attributed to the synergistic effects of the high solubility of the material, the high Mg^2+^ concentration perceived by the osteoblasts, and the serum protein-mediated apatite-like mineralization formed on the material. Both amorphous and crystalline TMP supported extensive monocyte proliferation but suspended osteoclast formation, indicating the promising potential of TMP, in particular the amorphous form, for preparing bone repair materials.

Multiple studies have suggested that magnesium favors bone regeneration in comparison to pure CaPs. Osteoblast proliferation was improved in brushite-based CPCs when substituted with Mg^2+^ [200]. Wu et al. demonstrated that the attachment and proliferation rate of MG-63 osteoblast-like cells was significantly higher for Mg-substituted CPCs compared to pure CPC and MPC [180]. The same type of cells was tested with Ca-Mg phosphate scaffolds: their attachment and proliferation, as well as the alkaline phosphatase (ALP) activity, were significantly enhanced in Mg-containing formulations in comparison with the pure Ca-based formulation [201]. Ewald et al. compared Ca-deficient hydroxyapatite, brushite, and STR cements and found that the cell activity normalized to the cell number was higher for brushite and STR when compared to hydroxyapatite [141]. A work from the same research group compared the passive and active in vitro resorption behavior of brushite, monetite (CaHPO_4_), Ca-deficient hydroxyapatite, and STR cements, finding the highest passive and active degradation behavior for the STR-based material [142].

Focusing on cell behavior for pure MPCs, many reports have described their excellent biocompatibility. A recent study from Liu et al., who developed a TMP-KH_2_PO_4_-based cement, showed that bone marrow mesenchymal stem cells (BMSCs) displayed high cell viability and better adhesion compared to a reference PMMA cement [160]. An MPC based on Mg(OH)_2_·MgCl_2_·8H_2_O as a binding phase demonstrated good biocompatibility towards rat bone marrow stromal cells, which showed good viability and attachment to the cement samples [77]. MPCs prepared with the aid of microwaves also showed good cytocompatibility towards MC3T3-E1 cells, comparable to that of a Ti scaffold [139]. A thorough study was carried out by Yu et al., who tested the inherited toxicology of an MPC prepared with MgO and NH_4_H_2_PO_4_, including a gene mutation assay (Ames test), chromosome aberration assay (micronucleus test), and DNA damage assay (unscheduled DNA synthesis test) [79]. The genetic results showed that MPC would cause neither an increase in the unscheduled DNA synthesis of human peripheral blood lymphocytes (i.e., no DNA damage), nor back mutation in murine typhoid salmonella (i.e., no gene mutations), revealing its nontoxic and safe nature. Human osteosarcoma cells (MG-63) were used to assess the biocompatibility of a MgO-based MPC prepared with KH_2_PO_4_, hydroxyapatite, sucrose, and borax [126]: cell proliferation and apoptosis were evaluated, demonstrating no cytotoxicity from the cements.

Concerning macroporous MPCs, Babaie et al. studied the viability and adhesion of MC3T3 osteoblast cells on a foamed macroporous MPC [69]. The porous cement showed significant stimulatory effects on cell proliferation and an improvement in differentiation, and this was attributed to the controlled release of Mg^2+^, which stimulated the cell response. The cells also attached and proliferated well on the sample surfaces, indicating no negative effects on cell morphology and viability. A different type of macroporous MPC obtained by foaming with calcium carbonate and citric acid was recently developed by Wang et al. and tested with hPDLSCs cells [117]. The cements demonstrated no significant cytotoxicity, and the cells could effectively adhere to the cements; in particular, one formulation was superior at controlling the MPC to promote cell growth and proliferation. The macroporous MPC foams developed by Ewald et al., based on TMP or STR prepared at different pH values, were tested for their cytocompatibility, and the results showed that the proliferation and cell activity of the osteoblasts was the highest for the TMP foams, followed by the STR foams fabricated at a pH of 8.5. The cell proliferation on the STR foams fabricated at a higher pH (10.5) was significantly reduced, probably due to the more alkaline nature of the material [143]. Another example of a macroporous MPC, prepared using NaCl granules as the templating agent, was treated in SBF before being inoculated with MC3T3-E1 cells [150]; the results showed that cells grew faster on the MPCs treated in SBF than on the scaffolds without SBF treatment until 7 days of culture, indicating that SBF modification positively supported the growth of MC3T3- E1 cells. Furthermore, the osteoblast differentiation of MC3T3-E1 cells increased with the SBF modification of the MgP scaffold, as demonstrated by the expression of osteoblast marker genes (ALP and osteocalcin).

As far as 3D-printed MPC scaffolds are concerned, Cao et al. exposed MC3T3-E1 osteoblast cells to extracts originating from NEW, STR, and brushite-based printed scaffolds [81]. All the tested systems showed good viability when in contact with the cement extracts. When seeding the cells onto cement scaffolds, differences were observed: osteoblasts could attach and spread well on the NEW and brushite scaffold surfaces, while almost no cells could be observed on the surface of the STR scaffold. The authors attributed this result to the strong alkaline environment produced by the STR scaffold degradation. In the work of Klammert et al., human osteoblastic cells were used to test the biocompatibility of a 3D-printed STR-based scaffold [140]: the cytocompatibility was satisfying, even though a reduced cell viability compared with the Ti and polystyrene controls was found. This was ascribed to the considerable solubility of the STR scaffold in a wet environment, as indicated by the significantly altered free electrolytes of the cell culture medium. These results seemed to suggest that the STR scaffold might not have had an optimal interaction with bone cells; nevertheless, other studies have affirmed the great potential of these materials. For instance, the 3D-printed STR scaffold developed by Lee et al. was inoculated with MC3T3-E1 cells, displaying good cell affinity and consequently promoting good cell proliferation, growth, and migration [146]. Key osteoblast genes, such as Coll-I, ALP, and OC, have also been evaluated to assess the effect of the differentiation process at the mRNA level: Coll-I is regularly used as an early marker of osteoblast differentiation; ALP is another early-stage marker of osteoblast differentiation that makes phosphate groups available for calcification; OC is secreted by osteoblasts and plays a role in mineralization and calcium ion homeostasis, being often used as a terminal marker of osteoblast differentiation. The upregulation of these bone-specific markers suggested the excellent osteogenic differentiation behavior of this scaffold.

Among the strategies to improve the biological performance of MPCs, the inclusion of different types of additives is a valuable technique. For instance, a metal organic framework, ZIF-8, could promote the viability, adhesion, spreading, and proliferation of mBMSCs (mouse bone marrow stem cells) [133]. The ALP activity and the expression of osteogenic differentiation-related genes (ALP, Coll-I, OCN, and OPN) by the cells on ZIF-MPC were significantly improved compared with the unmodified MPC. The inclusion of clay minerals could also improve the cell activity on MPCs [137]. As mentioned in Section 5.2, a novel indene compound was included in MPCs to improve their in vitro and in vivo performance [155]. The results showed that cells grew and adhered well on the scaffolds, with no significant growth retardation. The in vitro effect of the scaffolds on osteoblast differentiation was also examined by assessing the expression of osteoblast marker genes, proving the osteogenic activity of the material in vitro in a dose-dependent manner.

As far as MPCs modified with polymeric additives are concerned, MPCs prepared with different amounts of chondroitin sulfate have been thoroughly tested in terms of bioactivity via both in vitro and in vivo experiments [108]. The number of MC3T3-E1 cells on each cement sample increased over time, with all chondroitin sulfate-MPC samples showing higher values than MPC samples without polymers. In terms of cell adhesion, spreading, and morphology, all samples showed satisfying results. The ALP activity, which is an important indicator of osteoblast activity during osteogenic differentiation, was also evaluated. This parameter rapidly increased almost linearly with the increase in chondroitin sulfate content over 7 and 14 days. Overall, the inclusion of chondroitin sulfate in the MPCs promoted pre-osteoblast cell proliferation, attachment, and differentiation in vitro. A study from the same research group assessed the effect of carboxymethyl chitosan inclusion in MPCs [109]: pre-osteoblast MC3T3-E1 cells showed significantly greater adherence, proliferation, and differentiation on MPCs modified with CMC compared to the unmodified MPC. These cements were also demonstrated to effectively increase the adsorption of fibronectin and integrin-FAK-ERK signaling activation. When CMC was combined with alginate, the polymer-MPC composite was found to promote the attachment and proliferation of osteoblast cells and induce osteogenic differentiation in vitro, as verified by the expression of osteogenic markers [138]. A similar polymer, oxygen-CMC, also improved the proliferation, adhesion, and osteogenesis-related differentiation of MC3T3-E1 cells on MPCs [132]. Babaie et al. included PVA in amorphous magnesium-phosphate-based cements, in which CAT was the formed binding phase [164]. All the tested samples were biocompatible and did not show any cytotoxicity towards MC3T3-E1 cells, which could also attach and proliferate. None of the tested compositions suppressed the expression of osteoblast genes in MC3T3-E1, meaning that the cells could remain functional. The cited studies demonstrate that the inclusion of polymeric additives in MPCs might be a feasible strategy to not only improve the rheological properties of cement pastes (see Section 5.3), but also promote osteoblast interaction with the cement matrixes.

As a final note, it is worth mentioning that specific MPC formulations have displayed intrinsic antimicrobial activity: the works of Mestres et al. demonstrated that Na-containing MPCs had antibacterial activity towards *S. sanguinis* [57] and *E. coli*, *P. aeruginosa* (planktonic and in biofilm), and *A. actinomycetemcomitans*, which are strains associated with infected implants and hard-tissue-related infections [63]. More specifically, Na-MPC showed bactericidal properties (i.e., killed the bacteria), while NH_4_-MPC and NH_4_ + Na-MPC had a bacteriostatic effect (i.e., prevented their growth). This antimicrobial effect was attributed to a synergistic effect between the high osmolarity and alkaline pH of the MPC. This type of antimicrobial MPC could be of interest for application at sites with a high risk of bacteria formation, such as in maxillofacial surgery and endodontic treatments.

### 6.2. In Vivo Experiments

Although the use of MPCs in the orthopedic field can be considered in its early stages, many studies have already proven the in vivo potential of these materials. One of the first works in this sense was carried out by Yu et al., who studied the behavior of an MgO-based MPC in rabbits [79]. From macroscopic observations, no foreign body reaction, inflammation, or necrosis were found in vivo. A histological evaluation confirmed that the MPC formed direct bonds with the host bone, and the degradation behavior was satisfying.

Porous MPCs also display favorable features for bone regeneration: Wang et al. recently demonstrated that an MPC foamed with CaCO_3_ and citric acid implanted in rats mandibles could enhance bone regeneration [117]. Macroporous MPCs prepared with an NaCl template demonstrated promising in vivo performance: implantation into rabbit calvarial defects was studied at 4 and 8 weeks by means of micro-CT (micro-computed tomography) and histologic analyses. The porous scaffolds were degraded completely at 4 weeks with simultaneous bone and marrow-like structure regeneration, indicating an association between the rate of degradation and bone regeneration. Enhanced calcification compared to the nonporous MPC scaffold was also observed [150].

As already mentioned in the previous section, polymers are often included in MPCs to improve their performance. MPCs prepared with chondroitin sulfate were tested in rats’ parietal bones [108]: after 3 months, it was shown that the newly formed bone was larger and tighter in the cement prepared with chondroitin sulfate compared to pristine MPC, suggesting the importance of this polymer in enhancing bone formation after implantation. This effect was attributed to the binding of chondroitin sulfate to the extracellular matrix components, which allowed for the mediation of the binding of bone-like cells to the matrix, capturing soluble molecules such as growth factors into the matrix and at the cell surface. An MPC prepared with CMC-alginate was found to increase bone regeneration in a rat calvaria defect model compared to MPC alone [138].

An indene compound was included in a 3D-printed MPC and studied in a rat calvarial defect model [155]. After 4 and 8 weeks, micro-CT was used to study the formation of new bone, and it was observed that newly formed bone was increased 1.7-, 1.9-, and 2.3-fold for MPCs with 0, 5, and 25 µM indene compared with the empty hole, respectively.

Instead of including active molecules or polymers to promote the bioactivity of MPCs, Kaiser et al. recently tried to reduce the P/L ratio in the design of STR and KST cements to further improve the degradation speed [157]. Implants in sheep tibiae defects were studied after 2 and 4 months. Both cements were partially degraded and replaced by bone tissue, and the degradation speed of the KST was significantly higher compared to that of the STR. Whereas the KST cement might require further modification to achieve slower resorption and a reduced inflammatory response in vivo, the STR-based MPC appeared promising due to its constant degradation with simultaneous new bone formation.

It is also interesting to compare the effect of Mg inclusion in CPC, or the performance of MPCs when compared to CPCs. While studying brushite CPCs substituted with Mg, the work of Cabrejos-Azama showed that Mg-CPCs promoted new bone formation in comparison to CPCs in the rabbit calvaria [200]. Other studies have compared the in vivo performance of MPCs with that of CPCs: for instance, Kanter et al. implanted brushite, hydroxyapatite, and STR cements into sheep bones and monitored the process for 10 months [41]. No indication for wound infections, inflammation, or rejection was observed when the implants were retrieved after 4, 7, and 10 months. The size of the hydroxyapatite cement implant was unchanged even after 10 months, indicating the absence of degradation, and brushite was clearly visible with only a small reduction in the original size. In contrast, struvite cements were strongly degraded during the same period. Of note, micro-CT revealed that the defect was filled with mineralized tissue, which was similar to the surrounding bone. In a similar work, Klammert et al. compared, after 15 months of implantation in rat femurs, scaffolds based on CPCs (brushite and hydroxyapatite) and MPCs (NEW and STR) [202]. Micro-CT revealed the partial degradation of all the implants except hydroxyapatite. Zhang et al. compared the performance of a KST-based MPC with that of a CPC [125]: upon implantation in rabbits, no foreign body reaction, inflammation, or necrosis were observed. Both cements were resorbed and replaced by the newly formed bone in vivo, and the MPC displayed a higher resorption rate and enhanced bone regeneration compared to the CPC.

In the work of Zeng et al., an MPC, a CPC, and a mixed CMPC were used to perform a maxillary sinus floor elevation in rabbits: interestingly, the CMPC could promote more effectively new bone formation and mineralization compared to the reference CPC and MPC [203].

**Figure 8:**
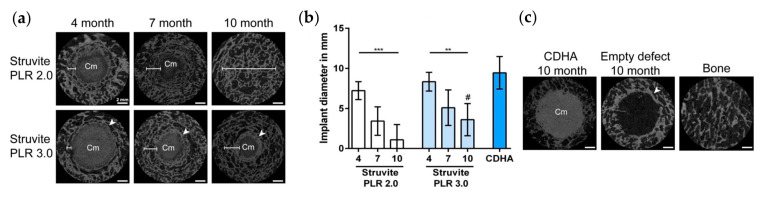
(**a**) Representative micro-CT images of STR femoral implants with a P/L ratio of 2.0 g/mL and 3.0 g/mL. Bars indicate the region around the remaining cements where new bone was formed. (**b**) Visible implant diameter after 4, 7, and 10 months of implantation (Levels of significance: ^**^ *p* < 0.01, ^***^ *p* < 0.001, ^#^ *p* < 0.05). (**c**) Representative micro-CT images of Ca-deficient hydroxyapatite (CDHA) implant and empty bone defect after 10 months, and intact bone from the same location. Arrowheads indicate sclerotic seams that formed near the struvite surface and at the edge of the empty defect, where the bone was denser. Adapted with permission from Ref. [154]. Copyright © 2023. Elsevier.

Kanter et al. compared the performance of STR-based MPCs prepared at two different P/L ratios (2 and 3 g/mL) with a Ca-deficient hydroxyapatite scaffold implanted into trabecular bones of ovine models [154]. The implants were analyzed after 4, 7, and 10 months: the micro-CT results (reported in Figure 8) revealed that STR dissolved completely during an implantation period of 10 months and was replaced by new trabecular bone. A higher P/L ratio led to slightly faster resorption due to an increased STR:TMP ratio. Of note, the Ca-deficient hydroxyapatite control cements did not significantly degrade, and the empty defects were not filled with bone after 10 months.

Finally, the commercial OsteoCrete formulation has been tested in a variety of animal models: Schendel et al. compared the performance of OsteoCrete with a commercial CPC for the repair of critically sized skull defects and cementing bone flaps in rabbits. Both materials were successful, but OsteoCrete showed a faster resorption and bone replacement rate than the CPC control [204]. A different study on a horse animal model evaluated the biomechanical strength, interface quality, and bone healing in bone–implant interfaces treated with OsteoCrete vs. CPC and PMMA-based cement [205]. The results showed that OsteoCrete promoted bone–implant bonding and adjacent bone osteogenesis, which may reduce the risk of screw loosening. Dental applications were also reported for OsteoCrete, but in this case, although the cement was successful in stabilizing the immediate dental implant in a large extraction socket when placed in a closed environment in the dog model, no benefits when compared to the controls were observed [206]. Besides the reported promising performance in animal models, clinical applications of this formulation have also been successful: the producers announced in 2020 that they had surpassed 1000 cases of implanting OsteoCrete^®^ in the US in various orthopedic applications.

## 7. Conclusions

This review summarized the state of the art for MPC applications in the biomedical field, in particular for bone repair. The interest in this type of material is rapidly growing, as shown by the number of recent publications that were discussed herein. The success of MPCs is driven by their attractive features in terms of setting time, injectability, degradation rate, and biocompatibility. Many reports have already demonstrated that in vivo they are able to be replaced by newly formed bone, providing a valuable strategy to fill bone voids and stabilize fractures. Notwithstanding the potentialities of MPCs, several challenges are still present, and there is plenty of room for improvement. In particular, novel strategies could be explored to further boost their performance, also taking inspiration from the well-established field of CPCs. For instance, it would be interesting to test other polymeric additives to improve the cohesion, anti-washout properties, and injectability of MPC pastes. Some promising examples could include polysaccharides such as hyaluronate and alginate, or modified celluloses. Moreover, the mechanical properties of MPCs might also be further improved by including biocompatible fibers. The possibility of loading bioactive molecules in MPCs that could be released upon application and promote bone regeneration is another field that has been neglected in the literature, but which could in principle greatly improve the biological performance of MPCs and contribute to opening up new application fields.

## Figures and Tables

**Table 1:** Magnesium phosphates: chemical formulae, mineral names, solubility product constants at 25 °C, and Mg/P atomic ratios.

Chemical Formula	Mineral Name	Solubility (K_sp_)	Mg/P Atomic Ratio	Abbreviation
Mg_3_(PO_4_)_2_	Farringtonite	3.9 × 10^−23^ [50]	1.5	TMP
Mg_3_(PO_4_)_2_·8H_2_O	Bobierrite	6.3 × 10^−26^ [51]	1.5	BOB
Mg_3_(PO_4_)_2_·22H_2_O	Cattiite ^1^	8 × 10^−24^ [51]	1.5	CAT
MgNH_4_PO_4_·H_2_O	Dittmarite	Unknown	1	DIT
MgHPO_4_·3H_2_O	Newberyite	1.5 × 10^−6^ [51]	1	NEW
MgHPO_4_·7H_2_O	Phosphorrösslerite	9.77 × 10^−18^ [50]	1	PHO
MgNH_4_PO_4_·6H_2_O	Struvite	7.1 × 10^−14^ [52]	1	STR
MgKPO_4_·6H_2_O	K- Struvite	2.4 × 10^−11^ [52]	1	KST
(NH_4_)_2_Mg_3_(HPO_4_)_4_·8H_2_O	Hannayite ^2^	Unknown	0.75	HAN
(NH_4_)_2_Mg(HPO_4_)_2_·4H_2_O	Schertelite ^2^	Unknown	0.5	SCH

^1^ This phase is metastable in water and converts to bobierrite [51]. ^2^ Dissolves incongruently in water with the formation of struvite [53].

**Table 2:** Comprehensive list of MPC formulations reported in the literature for applications in the biomedical and construction fields. In the Reference column, the former are marked with “B”, the latter with “C”, and those marked with “N” did not explicitly state the application field. Formulations containing Ca-doped MPCs or Mg-doped CPCs were not included in the list. The papers listed are those resulting from a search conducted in Web of Science using the keywords “magnesium phosphate cements” in the “topic” section.

Powder Component	Phosphate Salt Aqueous Solution	Additional Components	P/L ^1^	Final Crystalline Phases	References
MgO	NH_4_H_2_PO_4_	-	-	STR + SCH + DIT	[54]—N
MgO	NH_4_H_2_PO_4_	Silica	8.3–20 g/mL	MgO + STR	[55]—N
MgO	NH_4_H_2_PO_4_	Sand	4 g/mL	MgO + STR	[56]—C
MgO	NH_4_H_2_PO_4_ + NaH_2_PO_4_	Borax	7.7 g/mL	MgO + STR + SCH	[57]—B
MgO	NH_4_H_2_PO_4_ + NaH_2_PO_4_	Borax, Bi_2_O_3_	7.7 g/mL	MgO + STR + SCH	[58]—B
MgO	NH_4_H_2_PO_4_	Borax + Zn + quartz	5.6–7.1 g/mL	-	[59]—C
MgO	NH_4_H_2_PO_4_ + NaH_2_PO_4_	Chitosan	9 g/mL	MgO + STR + SCH + Na_2_Mg(HPO_4_)_2_	[60]—B
MgO	NH_4_H_2_PO_4_	Na_2_B_4_O_7_·5H_2_O	8.3 g/mL	MgO + STR + DIT	[61]—N
MgO	NH_4_H_2_PO_4_	Borax, acetic acid	8.3 g/mL	MgO + STR + Mg(acetate) + NH_4_H_2_PO_4_	[62]—C
MgO	NH_4_H_2_PO_4_ + NaH_2_PO_4_	Borax	7.7 g/mL	MgO + STR + SCH	[63]—B
MgO	NH_4_H_2_PO_4_	Borax, acetic acid, sand	8.3 g/mL	MgO + STR	[64]—C
MgO	NH_4_H_2_PO_4_	Borax, sand, metakaolin	4.3 g/mL	MgO + STR	[65]—C
MgO	NH_4_H_2_PO_4_	Borax, Na_5_P_3_O_10_, metakaolin, sand, sodium polyacrylate	5 g/mL	STR	[66]—C
MgO	NH_4_H_2_PO_4_	Borax, Na_5_P_3_O_10_, acetic acid	6.66 g/mL	MgO + STR	[67]—C
MgO	NH_4_H_2_PO_4_	H_3_BO_3_	-	MgO + STR + DIT	[68]—N
MgO + AMP	PVA solution	Mg granules	0.5 g/mL	BOB	[69]—B
MgO	NH_4_H_2_PO_4_	Na_5_P_3_O_10_, H_3_BO_3_, sand	5.3–6.3 g/mL	-	[70]—C
MgO	NH_4_H_2_PO_4_	Na_5_P_3_O_10_, borax, H_3_BO_3_, sand	-	-	[71]—C
MgO	NH_4_H_2_PO_4_	Metakaolin, Al_2_O_3_, borax, sand	11.1 g/mL	MgO + STR + Al phosphate hydrates	[72]—C
MgO	NH_4_H_2_PO_4_	Borax, citric acid, quartz, foaming agent	5.5–7.1 g/mL	MgO + STR + SiO_2_	[73]—C
MgO	NH_4_H_2_PO_4_	H_3_BO_3_	1.7 g/mL	MgO + STR	[74]—N
MgO	NH_4_H_2_PO_4_	Fly ash, sand, borax, Na_5_P_3_O_10_	5 g/mL	MgO + STR	[75]—C
MgO	NH_4_H_2_PO_4_	Borax, H_2_O_2_, Al_2_O_3_, zeolite	2.1–2.6 g/mL	MgO + STR + NH_4_H_2_PO_4_	[76]—N
MgO	H_3_PO_4_	MgCl_2_	0.6–1 g/mL	Mg(OH)_2_·MgCl_2_·8H_2_O	[77]—B
MgO	KH_2_PO_4_ +NH_4_H_2_PO_4_	Fly ash, borax, Na_5_P_3_O_10_,	10 g/mL	MgO + STR + KST	[78]—C
MgO	NH_4_H_2_PO_4_	retarder	-	-	[79]—B
MgO	KH_2_PO_4_, NH_4_H_2_PO_4_	Borax	7.1 g/mL	MgO + STR/KST + DIT	[80]—C
MgO	KH_2_PO_4_, NH_4_H_2_PO_4_	Pluronic F127	-	MgO, Mg(OH)_2_, STR, NEW	[81]—B
MgO	KH_2_PO_4_ + NH_4_H_2_PO_4_	Al_2_O_3_, borax	6.25–7.1 g/mL	MgO + STR + KST + alumina phosphate hydrate	[82]—N
MgO	KH_2_PO_4_ + NH_4_H_2_PO_4_	H_3_BO_3_, Na_5_P_3_O_10_	-	MgO + KST + KH_2_PO_4_ + lünebergite	[83]—C
MgO	KH_2_PO_4_, NaH_2_PO_4_	Borax, sand	5 g/mL	MgO + KST + NaST	[84]—C
MgO	KH_2_PO_4_	Borax	8.3–12.5 g/mL	MgO + KST + borax	[85]—C
MgO	KH_2_PO_4_	Borax	5–7.1 g/mL	MgO + KST	[86]—C
MgO	KH_2_PO_4_	Borax	0.1–0.4 g/mL	MgO + KST	[87]—C
MgO	KH_2_PO_4_	-	6.66 g/mL	MgO + KST	[88]—C
MgO	KH_2_PO_4_	Borax	-	MgO + KST	[89]—C
MgO	KH_2_PO_4_	Fly ash, H_3_BO_3_	3.6–3.8 g/mL	MgO + KST + SiO_2_	[90]—C
MgO	KH_2_PO_4_, Na_2_HPO_4_·12H_2_O	Borax	-	MgO + KST + Na_2_Mg(HPO_4_)_2_ + K_2_Mg(HPO_4_)_2_·4H_2_O	[91]—C
MgO	KH_2_PO_4_	Fly ash, borax, sand	5–7.1 g/mL	MgO + KST	[92]—C
MgO	KH_2_PO_4_	-	2–6.66 g/mL	MgO + KST	[93]—C
MgO	KH_2_PO_4_	-	3.3 g/mL	MgO + KST	[94]—N
MgO	KH_2_PO_4_	-	5 g/mL	MgO + KST	[95,96]—C
MgO	KH_2_PO_4_	Glucose	3.3–5.5 g/mL	MgO + KST	[97]—B
MgO	KH_2_PO_4_	-	2 g/mL	MgO + KST	[98]—C
MgO	KH_2_PO_4_	-	2.3 g/mL	MgO + KST	[99]—N
MgO	KH_2_PO_4_	-	2 g/mL	MgO + KST	[100]—N
MgO	KH_2_PO_4_	H_3_BO_3_	1 g/mL	Mg_2_KH(PO_4_)_2_·15H_2_O + KST	[101]—N
MgO	KH_2_PO_4_	Borax, fly ash, sand	4.3–6.2 g/mL	MgO + KST + SiO_2_	[102]—C
MgO	KH_2_PO_4_	Sand, fly ash,	3.3 g/mL	-	[103]—C
MgO	KH_2_PO_4_	Borax, NaHCO_3_	5.9 g/mL	MgO + KST	[104]—C
MgO	KH_2_PO_4_	Acylic latexes	2.3 g/mL	MgO + KST	[105,106]—C
MgO	KH_2_PO_4_	Silanes, borax, Na_5_P_3_O_10_	9 g/mL	MgO + KST	[107]—C
MgO	KH_2_PO_4_	Chondroitin sulfate	2 g/mL	MgO + KST	[108]—B
MgO	KH_2_PO_4_	Carboxymethyl chitosan	2 g/mL	MgO + KST	[109]—B
MgO	KH_2_PO_4_	-	0.2–4 g/mL	MgO + KST+ PHO + Mg_2_KH(PO_4_)_2_·15H_2_O	[110]—C
MgO	KH_2_PO_4_	Citric acid	2 g/mL	MgO + KST	[111]—N
MgO	KH_2_PO_4_	Borax, sand, sulphoaluminate cement	6.25 g/mL	MgO + KST + Ca-phases	[112]—C
MgO	KH_2_PO_4_	Borax, fly ash	7.14 g/mL	MgO + KST	[113]—C
MgO	KH_2_PO_4_	-	2.3 g/mL	MgO + KST	[114]—N
MgO	KH_2_PO_4_	Al_2_(SO_4_)_3_·16H_2_O	0.2–4 g/mL	MgO + KST + others	[115]—C
MgO	KH_2_PO_4_	Borax, Zn(NO_3_)_2_	0.1–7.14 g/mL	MgO + KST	[116]—C
MgO	KH_2_PO_4_	CaCO_3_, citric acid	0.16 g/mL	MgO + KST + CaCO_3_	[117]—B
MgO	KH_2_PO_4_	-	0.25–0.7 g/mL	MgO + KST	[118]—B
MgO	KH_2_PO_4_	Borax, fly ash, silica fume, sand	5.5 g/mL	-	[119]—C
MgO	KH_2_PO_4_	Borax, ferroaluminates	4.5 g/mL	MgO + KST + aluminates	[120]—C
MgO	KH_2_PO_4_	CaSiO_3_, quartz, MgCl_2_	1.2–4 g/mL	MgO + KST + CaSiO_3_ + Mg_2_KH(PO_4_)_2_·15H_2_O	[121]—C
MgO	KH_2_PO_4_	CaSiO_3_	4 g/mL	MgO + KST + BOB + MgKPO_4_·H_2_O + CaSiO_3_	[122]—C
MgO	KH_2_PO_4_	-	6.7 g/mL	MgO + KST + BOB	[123]—C
MgO	KH_2_PO_4_	Borax, limestone, Na_2_HPO_4_·12H_2_O	6.25 g/mL	MgO + KST + limestone	[124]—C
MgO	KH_2_PO_4_ + H_3_PO_4_	Sucrose, Na_5_P_3_O_10_, hydroxyapatite	3.3 g/mL	MgO + KST	[125]—B
MgO	KH_2_PO_4_	Sucrose, borax, hydroxyapatite	6.25 g/mL	MgO + KST	[126]—B
MgO	KH_2_PO_4_	-	6 g/mL	MgO + KST	[127]—B
MgO	KH_2_PO_4_	Borax, NaH_2_PO_4_	5 g/mL	Na-KST	[128]—C
MgO	KH_2_PO_4_	Borax, fly ash, sand, PVA fibers	5.3–10 g/mL	-	[129]—C
MgO	KH_2_PO_4_	Borax, sand, basalt	2.2 g/mL	MgO + KST + MgCO_3_ + MgCO_3_·3H_2_O	[130]—C
MgO	KH_2_PO_4_	Graphene oxide, borax, SDS	7.14 g/mL	MgO + KST	[131]—C
MgO	KH_2_PO_4_	Oxygen-carboxymethyl chitosan	2 g/mL	MgO + KST	[132]—B
MgO	KH_2_PO_4_	ZIF-8	2.5 g/mL	MgO + KST	[133]—B
MgO	KH_2_PO_4_, K_2_HPO_4_·3H_2_O	-	2.7 g/mL	MgO + KST+ MgKPO_4_·H_2_O	[134]—C
MgO	KH_2_PO_4_	H_3_BO_3_, polyurea aerogel	-	-	[135]—B
MgO	KH_2_PO_4_	H_3_BO_3_, Al(NO_3_)_3_	0.01 g/mL	CAT + NEW	[136]—C
MgO	KH_2_PO_4_	Laponite, sepiolite, halloysite	2 g/mL	MgO + KST	[137]—B
MgO	KH_2_PO_4_	Carboxymethyl chitosan, sodium alginate	2 g/mL	MgO + KST	[138]—B
Mg(OH)_2_	H_3_PO_4_	Microwaves	2.5 g/mL	NEW	[139]—B
TMP	(NH_4_)_2_HPO_4_ + NH_4_H_2_PO_4_	-	3 g/mL	TMP + STR + NEW	[140]—B
TMP + STR	(NH_4_)_2_HPO_4_	-	2.1 g/mL	TMP + STR	[141,142]—B
BOB	(NH_4_)_2_HPO_4_	TWEEN20, PU foam	-	TMP + STR	[143]—B
TMP	(NH_4_)_2_HPO_4_	(NH_4_)_2_C_6_H_6_O_7_	1–3.3 g/mL	TMP + STR	[144]—B
TMP	(NH_4_)_2_HPO_4/_K_2_HPO_4_/ H_3_PO_4_	HPMC	2 g/mL	STR, KST, NEW	[145]—B
TMP	(NH_4_)_2_HPO_4_ + NH_4_H_2_PO_4_	-	2–3 g/mL	TMP + STR	[46]—B
TMP	(NH_4_)_2_HPO_4_	HPMC	1.5 g/mL	TMP + STR	[146]—B
TMP	Phytic acid		3 g/mL	TMP + NEW	[147]—B
TMP	(NH_4_)_2_HPO_4_	-	1–2 g/mL	TMP + STR	[148]—B
TMP	(NH_4_)_2_HPO_4_ + NH_4_H_2_PO_4_	-	0.33–2 g/mL	HAN	[149]—B
TMP	(NH_4_)_2_HPO_4_	HPMC + NaCl	1.33 g/mL	TMP + STR	[150]—B
TMP ^2^	H_2_O	-	1.5 g/mL	TMP + CAT	[151]—B
TMP	(NH_4_)_2_HPO_4_	HPMC + gelatin	-	STR	[152]—B
TMP	(NH_4_)_2_HPO_4_	Halloysite	1.5 g/mL	TMP + STR	[153]—C
TMP	(NH_4_)_2_HPO_4_ + NH_4_H_2_PO_4_	-	2–3 g/mL	TMP + STR	[154]—B
TMP	(NH_4_)_2_HPO_4_	HPMC + indene	1.33 g/mL	TMP + STR	[155]—B
TMP	(NH_4_)_2_HPO_4_	ammonium citrate	2 g/mL	TMP + STR	[156]—B
TMP	(NH_4_)_2_HPO_4_ + NH_4_H_2_PO_4_/K_2_HPO_4_ + KH_2_PO_4_	-	0.6–1.5 g/mL	TMP + STR + NEW + SCH + KST	[157]—B
TMP	(NH_4_)_2_HPO_4_	Gelatin microparticles	1.5 g/mL	TMP + STR	[158]—B
TMP	(NH_4_)_2_HPO_4_	Borax	1.5 g/mL	TMP + STR	[159]—N
TMP	KH_2_PO_4_	-	2–3 g/mL	TMP + KST + NEW	[160]—B
MgO+ TMP	Phytic acid	-	2 g/mL	TMP + NEW	[161]—B
MgO+ TMP	Phytic acid	-	1.71–2 g/mL	MgO + TMP + NEW	[162,163]—B
AMP	H_2_O	PVA	0.4–0.6 g/mL	CAT	[164]—B

^1^ Powder/liquid ratio. When water/cement (w/c), water/solid (w/s), or water/binder (w/b) ratios were given in the paper, the reciprocal of that value, corresponding to the P/L ratio, is reported in the table. ^2^ Mechanically activated, also contains NaMg_4_(PO_4_)_3_ and quartz formed during milling.

**Table 3:** Magnesium content in the different tissues of a healthy adult [16].

Location	Mg Content (g)	% Mg in Total
Bone	12.720	60–65%
Muscle	6.480	27%
Other cells	4.608	6–7%
Erythrocites	0.120	0.5%
Serum	0.072	0.3%
Extracellular	-	<1%

## Data Availability

Not applicable.
